# Prevalence of a delayed diagnosis of caffeine poisoning in the emergency department of a Japanese hospital

**DOI:** 10.1002/jgf2.761

**Published:** 2024-12-03

**Authors:** Junpei Komagamine, Tomohiro Kurihara

**Affiliations:** ^1^ Department of Emergency and Critical Care Medicine NHO Tokyo Medical Center Tokyo Japan

**Keywords:** caffeine poisoning, metabolic acidosis, missed diagnosis

## Abstract

**Background:**

Most cases of acute caffeine poisoning are easily diagnosed at initial hospital visits. Nonetheless, accurate diagnosis is sometimes delayed in some patients with this condition. Therefore, our aim was to determine the prevalence of delayed diagnosis of acute caffeine poisoning in the emergency department (ED).

**Methods:**

A single‐center retrospective observational study was conducted. All consecutive patients with acute caffeine poisoning who were admitted to our hospital from January 2016 to March 2024 were included. The primary outcome was the proportion of patients who did not receive a correct diagnosis initially in the ED.

**Results:**

A total of 30 patients were included. The median age was 23 years (IQR 19–27), 24 (80%) were women, and 18 (60%) had psychiatric disorders. The diagnosis of acute caffeine poisoning was delayed in three patients (10%, 95% CI 0%–21%), among whom the initial diagnoses were encephalitis (*n* = 1), epileptic seizures (*n* = 1), and altered consciousness for unknown reasons (*n* = 1). Patients with delayed diagnoses experienced seizures more frequently during their ED stay than patients without delayed diagnoses did (*p* < 0.001).

**Conclusion:**

The initial diagnosis is missed in 1 of every 10 patients with acute caffeine poisoning. The presence of seizure as the initial sign was associated with delayed diagnosis of acute caffeine poisoning.

## Graphical abstract

The initial diagnosis is missed in 1 of every 10 patients with acute caffeine poisoning. The presence of seizure as the initial sign is associated with delayed diagnosis of acute caffeine poisoning.

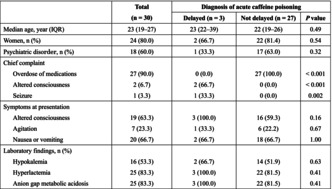

## 1 INTRODUCTION

Caffeine is a psychostimulant that is widely used to treat physical and mental fatigue.^1^ Because caffeine is included in many over‐the‐counter products, such as coffee and energy drinks, many people consume it daily.^2^ Although the intake of an optimal dose of caffeine is reported to be associated with several beneficial effects,^3^ caffeine overdose can result in serious illness and death. Owing to the easy availability of caffeine, cases of overdose can occur intentionally or unintentionally. In fact, the incidence of acute caffeine poisoning has increased in recent decades.^2^, ^4^

There are several therapies to combat acute caffeine poisoning,^5^, ^6^, ^7^, ^8^, ^9^, ^10^, ^11^, ^12^ although most of these treatments may be indicated regardless of the diagnosis. For example, when a young individual presents with a potentially fatal arrhythmia due to acute caffeine poisoning, venoarterial extracorporeal membrane oxygenation would be used even without the diagnosis of acute caffeine poisoning. However, recognizing acute caffeine poisoning enables clinicians to anticipate and manage potentially lethal conditions, such as arrhythmias, acidosis, or circulatory collapse. Therefore, it is crucial for patients with acute caffeine poisoning to be correctly diagnosed at their initial presentation because this would facilitate targeted interventions to prevent life‐threatening complications and improve overall outcomes.

Nonetheless, an accurate diagnosis of acute caffeine poisoning is sometimes difficult to make.^13^ Several fatal cases of acute caffeine poisoning in which a correct premortem diagnosis has not been made have also been reported.^14^, ^15^, ^16^ However, no studies have ever been conducted to evaluate the prevalence of a delayed diagnosis at initial presentation among patients with acute caffeine poisoning. In addition, few studies have investigated the clinical features and complications of this medical emergency.^4^, ^17^ Therefore, a retrospective observational study was conducted to evaluate clinical features and complications and determine the proportion of patients with a delayed diagnosis at the emergency department among patients who were hospitalized due to acute caffeine poisoning.

## 2 METHODS

### 2.1 Study design and setting

A retrospective observational study was conducted using the electronic medical records of the National Hospital Organization Tokyo Medical Center in Japan. The National Hospital Organization Tokyo Medical Center is a 600‐bed community acute care hospital located in Tokyo. Our hospital provides emergency care to approximately 7000 patients who arrive by ambulance annually. However, at our hospital, there are no attending physicians who are certified in toxicology.

Our research protocol was approved by the Medical Ethics Committee of the National Hospital Organization Tokyo Medical Center. We adhered to the ethical guidelines for epidemiological research in Japan and the Declaration of Helsinki. The need for individual informed consent was formally waived by the Medical Ethics Committee of the National Hospital Organization Tokyo Medical Center because we collected deidentified data without contacting the patients. However, in accordance with the Japanese Ethical Guidelines, we displayed an opt‐out statement in the waiting room and on the hospital's webpage to inform patients of the study and provide them the opportunity to refuse the use of their data.

### 2.2 The inclusion and exclusion criteria

The data of patients who were hospitalized because of acute caffeine poisoning in the Department of Emergency and Critical Care Medicine of our hospital from January 1, 2016, to March 31, 2024 were used. To minimize selection bias, all consecutive patients hospitalized with acute caffeine poisoning during the study period were screened. Patients who met all the following criteria were included: (1) The patient had ingested products containing ≥1000 mg of caffeine or their serum concentration of caffeine was greater than or equal to 20 μg/mL. (2) The principal diagnosis for hospitalization was acute caffeine poisoning. Patients who had concomitantly ingested a toxic dose of active ingredients other than caffeine were excluded.

### 2.3 Data collection and outcome measures

Information on patient age, gender, past medical history, caffeine dose, vital signs, clinical symptoms, treatment for acute caffeine poisoning, complications, and prognosis was retrospectively extracted from the electronic medical records. On the basis of a previous study,^4^ hyperthermia was defined as a body temperature of ≥37.5°C; tachypnea was defined as a respiratory rate of ≥20 breaths per minute, tachycardia was defined as a heart rate of ≥100 bpm, and a dilated pupil was defined as a pupil diameter of more than 4 mm. Liver injury was defined as an alanine aminotransferase level greater than 80 IU/L. Hypercreatine kinasemia was defined as a serum creatine kinase (CK) concentration greater than 200 IU/L, and rhabdomyolysis was defined as a serum CK concentration greater than 10,000 IU/L with myoglobinuria. Hypokalemia was defined as a serum potassium concentration of less than 3.5 mEq/L; hyperglycemia was defined as a serum glucose concentration greater than 180 mg/dL; and hyperlactemia was defined as a serum lactate concentration greater than 2 mmol/L. In accordance with previous guidelines,^18^ the pathological threshold of the serum anion gap (AG) was defined as a value greater than 12 mEq/L.

The primary outcome was the proportion of patients who received a delayed diagnosis of acute caffeine poisoning among all patients hospitalized due to acute caffeine poisoning. A delayed diagnosis was defined as occurring if a patient who was not diagnosed with acute caffeine poisoning at admission was later diagnosed with acute caffeine poisoning after admission.

### 2.4 Statistical analysis

The baseline characteristics and complications of the included patients were reported via descriptive statistics. For this information, the differences between patients with and without a delayed diagnosis of acute caffeine poisoning were evaluated via the chi‐square test or Mann–Whitney *U* test. The level of statistical significance was set at 5%. Stata version 15 (LightStone, Tokyo, Japan) was used to conduct these analyses.

## 3 RESULTS

During the study period, 58 patients were hospitalized at our hospital due to acute caffeine poisoning (Figure S1). Of those, 18 patients who consumed products with caffeine doses of less than 1000 mg were excluded. Nine patients were excluded due to concurrent overdose of acetaminophen. One patient was not included because he was not hospitalized. Thus, 30 patients were included in the final analysis.

Among the 30 included patients, the median age was 23 years (interquartile range (IQR) 19–27); 24 (80.0%) patients were women, and 18 (60.0%) patients had a history of psychiatric diseases (Table 1 and Table S1). The median estimated dose of caffeine intake was 3060 mg (IQR: 2135 mg to 5270 mg), and 10 patients (33.3%) had no concurrent intake of medications other than caffeine. Medication overdose was a chief complaint in 27 (90.0%) of all patients. On presentation, the most common symptoms were nausea or vomiting (*n* = 20, 66.7%) and altered levels of consciousness (*n* = 19, 63.3%), followed by agitation (*n* = 7, 23.3%). None of the patients had abdominal pain. Tachypnea, tachycardia, and hyperthermia were observed in 20 (69.0%), 16 (53.3%), and 4 (14.3%) patients, respectively. Regarding laboratory findings at presentation, hypokalemia, hyperglycemia, hypercreatine kinasemia, hyperlactemia, and anion gap metabolic acidosis were observed in 16 (53.3%), 11 (36.7%), 13 (43.3%), 25 (83.3%), and 25 (83.3%) patients, respectively.

**TABLE 1:** Characteristics of the 30 hospitalized patients with acute caffeine poisoning.

	Total(*n* = 30)	Diagnosis of acute caffeine poisoning		
		Delayed (*n* = 3)	Not delayed (*n* = 27)	*p* value^a^
Median age, year (IQR)	23 (19–27)	23 (22–39)	22 (19–26)	0.49
Women, *n* (%)	24 (80.0)	2 (66.7)	22 (81.4)	0.54
Median body mass index (IQR)	21.5 (18.5–23.4)	16.8 (16.5–18.8)	21.7 (19.0–23.5)	0.08
Psychiatric disorder, *n* (%)	18 (60.0)	1 (33.3)	17 (63.0)	0.32
Estimated caffeine intake, mg (IQR)	3060 (2135–5270)	2400 (1830–4700)	3120 (2170–5180)	0.58
Median time to presentation from symptom onset, hours (IQR)	4 (2–9)	2 (2–8)	5 (2–9)	0.52
Chief complaint				
Overdose of medications	27 (90.0)	0 (0.0)	27 (100.0)	<0.001
Altered consciousness	2 (6.7)	2 (66.7)	0 (0.0)	<0.001
Seizure	1 (3.3)	1 (33.3)	0 (0.0)	0.002
Symptoms during the ED stay				
Altered consciousness	19 (63.3)	3 (100.0)	16 (59.3)	0.16
Agitation	7 (23.3)	1 (33.3)	6 (22.2)	0.67
Nausea or vomiting	20 (66.7)	2 (66.7)	18 (66.7)	1.00
Headache	2 (6.0)	0 (0.0)	2 (7.4)	0.63
Seizure	3 (10.0)	3 (100.0)	0 (0.0)	<0.001
Neurological signs at presentation				
Median GCS scores (IQR)	15 (12–15)	7 (5–10)	15 (13–15)	0.02
Mydriasis, n (%)	3 (11.1)	0 (0.0)	3 (12.0)	0.33
Vital signs at presentation, n (%)				
Tachycardia	16 (53.3)	3 (100.0)	13 (48.2)	0.09
Tachypnea	20 (69.0)	3 (100.0)	17 (65.4)	0.43
Hyperthermia	4 (14.3)	1 (33.3)	3 (12.0)	0.52
Laboratory findings, *n* (%)				
Hypokalemia	16 (53.3)	2 (66.7)	14 (51.9)	0.63
Hyperglycemia	11 (36.7)	3 (100.0)	8 (29.6)	0.02
Hypercreatine kinasemia	13 (43.3)	3 (100.0)	10 (37.0)	0.04
Hyperlactemia	25 (83.3)	3 (100.0)	22 (81.5)	0.41
Anion gap metabolic acidosis	25 (83.3)	3 (100.0)	22 (81.5)	0.41

Abbreviations: ED, emergency department; GCS, Glasgow coma Scale; IQR, interquartile range.

^a^ Comparisons between patients with and without a delayed diagnosis of acute caffeine poisoning were performed via the chi‐square test or Mann–Whitney *U* test.

For the treatment of acute caffeine poisoning, activated charcoal, gastric lavage, ß‐blockers, and hemodialysis were used in 5 (16.7%), 2 (6.7%), 2 (6.7%), and 1 (3.3%) of the patients, respectively (Table 2). While tracheal intubation was needed in one patient (3.3%), extracorporeal membrane oxygenation was not used in any patients. Regarding complications, rhabdomyolysis and liver injury were observed in 2 (6.7%) and 3 (10.0%) patients, respectively. No patients died during hospitalization.

**TABLE 2:** Treatment and prognosis of the 30 patients with acute caffeine poisoning.

	Total (*n* = 30)	Diagnosis of acute caffeine poisoning		*p* value^a^
		Delayed (*n* = 3)	Not delayed (*n* = 27)	
Treatment, *n* (%)				
Activated charcoal	5 (16.7)	1 (33.3)	4 (14.9)	0.41
Gastric lavage	2 (6.7)	0 (0.0)	2 (7.4)	0.63
ß‐blocker	2 (6.7)	0 (0.0)	2 (7.4)	0.63
Tracheal intubation	2 (6.7)	1 (33.3)	1 (3.7)	0.05
Dialysis	1 (3.3)	0 (0.0)	1 (3.7)	0.73
Complications, *n* (%)				
Liver injury	3 (10.0)	1 (33.3)	2 (7.4)	0.16
Rhabdomyolysis	2 (6.7)	1 (33.3)	1 (3.7)	0.05
Gastrointestinal bleeding	1 (3.3)	0 (0.0)	1 (3.7)	0.73
Necrotizing esophagitis	1 (3.3)	1 (3.3)	0 (0.0)	0.002
Median days of hospital stay (IQR)	2 (1–4)	11 (8–13)	1 (1–3)	0.02
Discharge to home, *n* (%)	28 (93.3)	2 (66.7)	26 (96.3)	0.05
In‐hospital death, *n* (%)	0 (0.0)	0 (0.0)	0 (0.0)	1.00

Abbreviation: IQR, interquartile range.

^a^ Comparisons between patients with and without a delayed diagnosis of acute caffeine poisoning were performed via the chi‐square test or Mann–Whitney *U* test.

As for the primary outcome, a delayed diagnosis of acute caffeine poisoning occurred in 3 (10.0%) of the patients. Compared with patients without delayed diagnosis, those with a delayed diagnosis were more likely to have seizure, hyperglycemia, and hypercreatine kinasemia. Furthermore, the Glasgow coma scale scores were significantly lower in patients with delayed diagnoses than in those without. Among the three patients with delayed diagnoses, their initial diagnoses at the emergency department were altered levels of consciousness of unknown cause (*n* = 1), epileptic seizure (*n* = 1), and acute encephalitis (*n* = 1). Moreover, anion gap metabolic acidosis and hyperlactemia were observed in all three patients.

## 4 DISCUSSION

Our findings revealed that the initial diagnosis was missed at the ED in one of every 10 patients hospitalized due to acute caffeine poisoning. Moreover, the presence of seizure as the initial sign was associated with delayed diagnosis of acute caffeine poisoning.

To our knowledge, the present study is the first to determine the prevalence of delayed diagnosis of acute caffeine poisoning in the ED. In the present study, an initial diagnosis at the ED was missed in a substantial proportion of patients with acute caffeine poisoning. Although no patients died in this study, given that early accurate diagnosis of acute caffeine poisoning can prevent life‐threatening complications, the prevalence of delayed diagnosis seems problematic. Therefore, further studies to determine the prevalence of delayed diagnosis for acute caffeine poisoning at other hospitals are warranted. Moreover, additional efforts to avoid a missed diagnosis of acute caffeine poisoning are needed.

Caffeine is a nonselective adenosine receptor antagonist that induces catecholamine secretion,^1^ which is associated with lactic acidosis.^19^, ^20^ Therefore, patients with acute caffeine poisoning often present with adrenergic toxidrome^21^ and anion gap metabolic acidosis. However, the symptoms associated with adrenergic toxidrome are sometimes not fully exhibited by patients with acute caffeine poisoning,^22^ as was also indicated by the present findings. In the present study, the laboratory test results of all patients with delayed diagnosis of acute caffeine poisoning revealed lactic acidosis. Therefore, the consideration of acute caffeine poisoning as a differential diagnosis of anion gap metabolic acidosis can help clinicians avoid a missed diagnosis of this medical emergency. However, in the mnemonic regarding the causes of anion gap metabolic acidosis, lactic acidosis but not caffeine poisoning is included in the list of differential diagnoses.^23^, ^24^ Moreover, theophylline or beta agonists are included in the list of differential diagnoses of drug‐induced lactic acidosis.^19^, ^25^ Although caffeine is a beta agonist and theophylline is a byproduct of caffeine, it is possible that clinicians cannot distinguish caffeine from beta agonists and theophylline. In addition, the presence of seizure as the initial sign might cause clinicians not to consider the differential diagnosis of lactic acidosis because the epileptic seizure can be deemed the cause of the lactic acidosis.^26^ Thus, to avoid a missed diagnosis of acute caffeine poisoning, caffeine poisoning should be considered in the differential diagnosis for patients with seizure and lactic acidosis of unknown cause.

In the present study, the most common symptoms were nausea or vomiting, followed by altered levels of consciousness and agitation. Regarding vital signs, tachypnea and tachycardia were reported in more than half of the patients with acute caffeine poisoning. These results are similar to those of a previous survey study.^4^ Although abdominal pain was reported to be a common symptom in another past investigation,^17^ abdominal pain was not reported in any of the patients in the present study or in the previous survey study.^4^ Most patients included in the past investigation^17^ were alert, whereas more than one‐third of patients included in the latter two studies had altered levels of consciousness. Therefore, the difference in the severity of acute caffeine poisoning might explain the discrepancy in the results for clinical symptoms between the present study and past investigation.^17^ The frequencies of liver injuries and rhabdomyolysis due to acute caffeine poisoning in the present study were also similar to those reported in the previous survey study.^4^ Although the previous study had a risk of selection bias because it was a survey study,^4^ our findings support its results.

Several limitations should be mentioned. First, our study had a retrospective observational design. Therefore, the collected data might be inaccurate. Second, the sample size of our study was small. Moreover, it was a single‐center investigation. Therefore, further multicenter studies are needed to validate our results. Third, we did not routinely measure the serum caffeine concentration in all patients. Finally, undiagnosed cases of fatal caffeine poisoning occurring during the study period might have been missed. Therefore, the prevalence of delayed diagnosis for acute caffeine poisoning might be underestimated.

In conclusion, the diagnosis of acute caffeine poisoning is initially missed in a substantial proportion of patients with this condition in the ED. The presence of seizure as the initial sign was associated with delayed diagnosis of acute caffeine poisoning.

## CONFLICT OF INTEREST STATEMENT

The authors declare no conflicts of interest.

## ETHICS STATEMENT

None.

## Supporting information


Data S1.
