# Supplementary material for: Prevalence of a delayed diagnosis of caffeine poisoning in the emergency department of a Japanese hospital
Source: J Gen Fam Med. 2024 Dec 3;26(2):157–62. doi: 10.1002/jgf2.761 (PMC11890057; doi:10.1002/jgf2.761)
Supplement: Supplementary file 1 — Data S1. [file JGF2-26-157-s001.docx]

A total of 58 patients were diagnosed with caffeine poisoning.

Twenty-eight patients were excluded:

- Caffeine consumption of less than 1000 mg (n = 18)
- Concurrent overdose of acetaminophen (n = 9)
- Was not hospitalized (n = 1)

Thirty patients (n=30) were included in the final analysis.

**Figure S1.** Flow chart of the 30 patients included in this study.

**Table S1.** Characteristics of the 30 hospitalized patients with acute caffeine poisoning.

|  | **Total**  **(n = 30)** | **Diagnosis of acute caffeine poisoning** | |
| --- | --- | --- | --- |
|  |  | **Delayed (n = 3)** | **Not delayed (n = 27)** |
| Age, years  Median (IQR)  Mean (SD) | 23 (19–27)  27.1 (13.5) | 23 (22–39)  32.7 (19.4) | 22 (19-26)  26.5 (13.0) |
| Women, n (%) | 24 (80.0) | 2 (66.7) | 22 (81.4) |
| Japanese ethnicity, n (%) | 29 (96.7) | 3 (100.0) | 26 (96.3) |
| Median body mass index (IQR) | 21.5 (18.5–23.4) | 16.8 (16.5–18.8) | 21.7 (19.0–23.5) |
| History of psychiatric disorders | 18 (60.0) | 1 (33.3) | 17 (63.0) |
| Estimated dose of caffeine intake  Median (IQR)  Mean (SD) | 3060 (2135–5270)  4738 (4218) | 2400 (1830–4700)  3553 (3039) | 3120 (2170–5180)  4870 (4354) |
| No concurrent intake of medications other than caffeine, n (%) | 10 (33.3) | 1 (33.3) | 9 (33.3) |
| Symptoms during the ED stay, n (%)  Altered consciousness  Agitation  Nausea or vomiting  Headache  Dizziness  Abdominal pain  Diarrhea  Seizure | 19 (63.3)  7 (23.3)  20 (66.7)  2 (6.0)  2 (6.0)  0 (0.0)  1 (3.3)  3 (10.0) | 3 (100.0)  1 (33.3)  2 (66.7)  0 (0.0)  0 (0.0)  0 (0.0)  0 (0.0)  3 (100.0) | 16 (59.3)  6 (22.2)  18 (66.7)  2 (7.4)  2 (7.4)  0 (0.0)  1 (3.7)  0 (0.0) |
| Neurological signs at presentation  Median GCS scores (IQR)  Mydriasis, n (%) | 15 (12–15)  3 (11.1) | 7 (5–10)  0 (0.0) | 15 (13–15)  3 (12.0) |
| Vital signs at presentation, mean (SD)  Heart rate, per minute  Systolic blood pressure, mmHg  Diastolic blood pressure, mmHg  Respiratory rate, per minute  Body temperature, °C | 108 (27)  116 (32)  78 (18)  23 (6)  36.6 (1.1) | 138.7 (27.4)  115.7 (27.4)  73.0 (7.5)  22 (3)  35.9 (3.3) | 104.5 (24.4)  116.6 (32.6)  78.4 (19.0)  22.8 (6.4)  36.6 (0.6) |
| Blood gas analysis, mean (SD)  pH  Bicarbonate, mmol/L  Anion gap, mmol/L  Lactate, mmol/L | 7.39 (0.13)  20.4 (5.2)  20.2 (6.1)  2.5 (2.0) | 7.15 (0.31)  13.4 (5.9)  30.4 (6.3)  6.5 (3.8) | 7.41 (0.07)  21.1 (4.7)  19.0 (5.0)  2.1 (1.2) |
| Laboratory tests, mean (SD)  White-cell count, per µL  Hemoglobin, g/dL  Platelet count, per µL  Total protein, g/dL  Albumin, g/dL  Alanine aminotransferase, U/L  Aspartate aminotransferase, U/L  Lactate dehydrogenase, U/L  Urea nitrogen, mg/dL  Creatinine, mg/dL  Sodium, mmol/L  Potassium, mmol/L  Chloride, mmol/L  Creatinine kinase, mg/dL  Glucose, mg/dL | 11697 (6344)  13.7 (1.7)  29.5 (6.7)  7.6 (0.7)  4.3 (0.7)  27.6 (42.3)  28.4 (15.3)  236.8 (90.1)  11.1 (8.1)  0.7 (0.3)  139.9 (1.9)  3.3 (0.7)  102.3 (2.7)  215.0 (332.7)  158.4 (59.0) | 21933 (3008)  14.7 (0.6)  36.4 (11.3)  8.1 (0.4)  N.A.  22.7 (19.4)  33.3 (15.0)  276.0 (59.8)  11.6 (5.8)  1.0 (0.3)  141.0 (1.0)  3.5 (1.0)  100.3 (2.5)  381.3 (418.6)  260.0 (90.4) | 10559 (5546)  13.6 (1.7)  28.8 (5.9)  7.5 (0.7)  4.3 (0.7)  28.2 (44.3)  27.9 (15.5)  232.5 (92.6)  11.0 (8.4)  0.7 (0.2)  139.8 (2.0)  3.3 (0.7)  102.5 (2.7)  196.6 (326.2)  147.1 (44.0) |
| Electrocardiograpy, mean (SD)  PR, minutes per second  QRS, minutes per second  QTc, minutes per second | 153.2 (19.1)  89.9 (9.6)  423.6 (34.1) | 130.7 (32.6)  88.0 (8.0)  416.7 (3.2) | 156.1 (15.6)  90.1 (9.9)  424.4 (36.1) |
| Serum caffeine concentration^a^, µg/mL  Median (IQR)  Mean (SD) | 60.0 (49.2–64.7)  61.4 (27.3) | 41.2 (34.1–48.3)  41.2 (20.0) | 64.7 (60.3–75.9)  71.4 (26.5) |

^a^The serum caffeine concentration was measured in only 7 patients.

ED, emergency department; GCS, Glasgow Coma Scale; IQR, interquartile range; SD, standard deviation.
